# A Case of Unresectable Advanced Esophageal Cancer with Recurrent Pneumatosis Intestinalis and Portal Venous Gas during Chemotherapy with Immune Checkpoint Inhibitors

**DOI:** 10.70352/scrj.cr.25-0603

**Published:** 2025-12-10

**Authors:** Hiroki Tashiro, Takafumi Suzuki, Hironori Tsujimoto, Seiichiro Fujishima, Hanae Shinada, Risa Kariya, Naoyuki Uehata, Asuma Ide, Keita Kouzu, Hiroyuki Horiguchi, Yoshihisa Yaguchi, Kaya Ijiri, Kosuke Miyai, Susumu Matsukuma, Hideki Ueno

**Affiliations:** 1 Department of Surgery, National Defense Medical College, Tokorozawa, Saitama, Japan; 2 Department of Laboratory Medicine, National Defense Medical College Hospital, Tokorozawa, Saitama, Japan

**Keywords:** esophageal squamous cell carcinoma, immune checkpoint inhibitors, immune-related adverse events, pneumatosis intestinalis, portal venous gas

## Abstract

**INTRODUCTION:**

Esophageal squamous cell carcinoma is an aggressive malignancy often diagnosed at an advanced stage. Immune checkpoint inhibitors (ICIs), such as pembrolizumab, have shown promising outcomes for improving survival. Although rare, immune-related adverse events (irAEs) associated with ICI therapy, such as pneumatosis intestinalis (PI) and portal venous gas (PVG), can be fatal.

**CASE PRESENTATION:**

A 60-year-old man with unresectable advanced esophageal squamous cell carcinoma received cisplatin, 5-fluorouracil (FP), and pembrolizumab. Three days after treatment initiation, the patient experienced abdominal pain and hypotension. Imaging revealed extensive PI and PVG, with no signs of bowel ischemia. Emergency laparotomy confirmed PI involving the entire length of the small intestine, with no visible perforations. Symptoms resolved with conservative management. After benefits and potential adverse events of the current chemotherapy regimen were explained, the patient chose to continue pembrolizumab-based chemotherapy. In the second and third courses, the doses of cisplatin and 5-FU were reduced to 75% (cisplatin 60 mg/m^2^, 5-FU 600 mg/m^2^), while the dose of pembrolizumab was maintained (200 mg). No major adverse events occurred, and follow-up CT scans showed shrinkage of the primary lesion, and PI did not recur during this period. However, 26 days after the 3rd treatment course, the patient developed sudden-onset abdominal pain, shock, more severe PI, and PVG. Despite receiving intensive care, the patient died the following day. Autopsy revealed diffuse intestinal erosion with a CD8-positive lymphocytic infiltration and histologically confirmed pneumatosis intestinalis extensively involving the small intestine and colon.

**CONCLUSIONS:**

The patient experienced two episodes of PI and PVG with distinct clinical courses. Although the exact cause of the first episode remains unclear, the fatal recurrence suggests that rechallenging with suspected causative agents carries a significant risk, even after dose reduction. While ICI-based chemotherapy for esophageal cancer was highly effective, this case highlights the importance of continuously reassessing the risk–benefit balance during treatment. Discontinuation of ICIs should be considered when serious irAE are suspected.

## Abbreviations

5-FU
: 5-fluorouracil
CRP
: C-reactive protein
ESCC
: esophageal squamous cell carcinoma
ICIs
: immune checkpoint inhibitors
irAE
: immune-related adverse events
PI
: pneumatosis intestinalis
PVG
: portal venous gas
WBC
: white blood cell count

## INTRODUCTION

Esophageal squamous cell carcinoma (ESCC) is a highly aggressive malignancy with poor prognosis, often diagnosed at an advanced stage.^1)^ Conventional treatment modalities, including surgery, chemotherapy, and radiation therapy have shown limited efficacy in improving long-term survival rates. Recently, immune checkpoint inhibitors (ICIs) have emerged as promising therapeutic options for various malignancies, including ESCC. It is reported that ICI-based chemotherapy improved overall survival of patients with unresectable advanced ESCC.^2,3)^

Pneumatosis intestinalis (PI) is a rare condition characterized by the presence of gas within the wall of the gastrointestinal tract.^4)^ The clinical presentation of PI varies widely, ranging from benign self-limiting conditions to a life-threatening emergency requiring immediate surgical intervention.^5)^ PI accompanied by portal venous gas (PVG) is potentially lethal, and necessitates urgent interventions.^6)^ The pathogenesis of PI remains controversial, implicating various underlying conditions, including chronic obstructive pulmonary disease, collagen vascular diseases, necrotizing enterocolitis, intestinal infections, ischemic bowel disorders, and immunosuppressive drug therapy.^4,7,8)^ Recently, PI has been recognized as a potential immune-related adverse event (irAE) associated with ICI therapy.^9–11)^

Here, we present a case of unresectable advanced ESCC, complicated by recurrent PI and PVG during chemotherapy with ICIs.

## CASE PRESENTATION

**Fig. 1:**
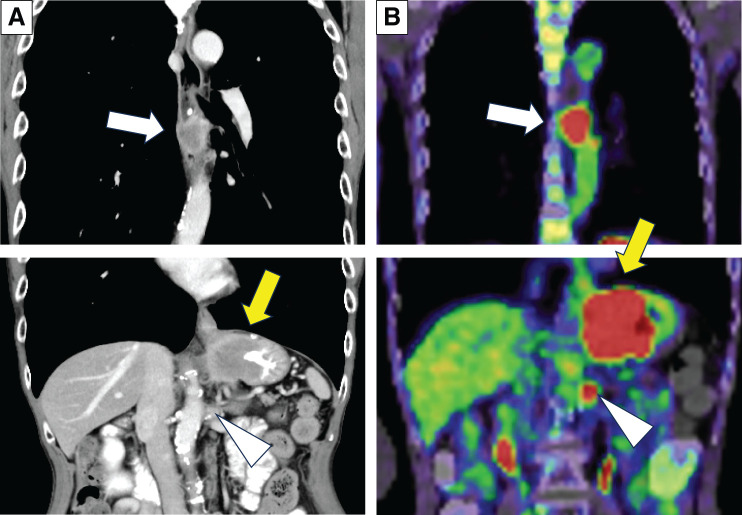
Images of CT, and PET-CT. CT showing an approximately 3- cm primary tumor in the middle esophagus (white arrows), along with enlarged para-aortic lymph node (arrow heads) and lymph nodes in the lesser curvature of the stomach (yellow arrows), which were suspected to have invaded the stomach wall (**A**). PET-CT showing increased uptake in these areas, suggesting metastases from esophageal cancer (**B**).

A 60-year-old man presented to our hospital with dysphagia. Upper gastrointestinal endoscopy revealed esophageal cancer, with stenosis in the middle thoracic esophagus, making it difficult for the endoscope to pass through. The tumor was histologically diagnosed as moderately differentiated squamous cell carcinoma. The levels of squamous cell carcinoma antigen and cytokeratin 19 fragments were both elevated, at 5.1 ng/mL (normal range: <3.5 ng/mL) and 30.0 ng/mL (normal range: <1.5 ng/mL), respectively. CT revealed an approximately 3 cm primary tumor in the middle esophagus, along with enlarged para-aortic and lesser curvature lymph nodes, which were suspected to have invaded the stomach wall (**Fig. 1A**). PET-CT scans revealed increased uptake in these areas, suggesting metastases from esophageal cancer. Based on these findings, the clinical diagnosis was classified as cT3cN2M1 cStage IVB esophageal cancer according to the Japanese Classification of Esophageal Cancer, 12th edition^12)^ (**Fig. 1B**). Laparoscopic jejunostomy was performed two weeks before initiating chemotherapy due to stenosis caused by the primary tumor, and enteral nutrition was initiated using a semi-digested formula.^13)^

**Fig. 2:**
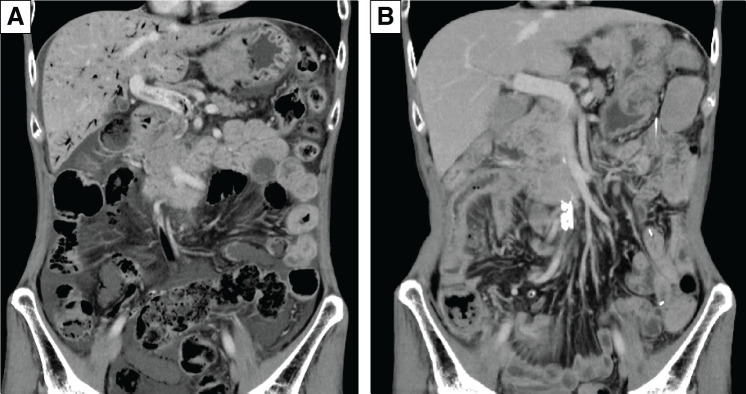
CT of the first occurrence of PI and PVG. Contrast-enhanced CT revealing extensive PI involving the entire length of the small intestine, and prominent gas extending from the mesenteric veins to the portal vein, with no evidence of free air or areas of poor contrast enhancement in the intestines (**A**). Five days after initiating chemotherapy, the patient’s abdominal symptoms and overall condition rapidly improved, and both PI and PVG signs were no longer visible on CT (**B**). PI, pneumatosis intestinalis; PVG, portal venous gas

Three days after initiating pembrolizumab (200 mg on day 1), cisplatin (80 mg/m^2^ on day 1), and 5-fluorouracil (5-FU) (800 mg/m^2^ on days 1–5), the patient complained of upper abdominal pain and bloating. Rebound tenderness in the upper abdomen and hypotension were also noted. Blood tests showed slightly elevated C-reactive protein (CRP) at 0.62 mg/dL and white blood cell count (WBC) at 10200/μL, whereas lactate level and base excess remained within normal range at 2.2 mmol/L and 2.9, respectively. A contrast-enhanced CT revealed extensive PI involving the entire length of the small intestine, as well as prominent gas extending from the mesenteric veins to the portal vein, with no evidence of free air or areas of poor contrast enhancement in the intestines (**Fig. 2A**). Due to the potentially life-threatening nature of PVG, chemotherapy was discontinued and an emergency laparotomy was performed.^6)^ During laparotomy, PI involving the entire length of the small intestine was confirmed. However, no evidence of gastrointestinal perforation or necrosis was observed. Postoperatively, the patient’s vital signs stabilized, enteral nutrition was discontinued, and antibiotics were administered. However, no steroids were given. Five days after initiating chemotherapy, the patient’s abdominal symptoms and overall condition rapidly improved, and both PI and PVG signs were no longer visible on CT (**Fig. 2B**). Enteral nutrition was resumed on day 7 using a fully digested formula. The patient was discharged on day 16, as there were no particular complaints, a colonoscopy was not performed.

After explaining both the potential benefits and potential risks associated with the resume of current chemotherapy regimen, the patient chose to proceed with the pembrolizumab-based chemotherapy regimen at a reduced dose. In the second and third courses, the doses of cisplatin and 5-FU were reduced to 75% (cisplatin 60 mg/m^2^, 5-FU 600 mg/m^2^), whereas the dose of pembrolizumab was maintained (200 mg). The treatment interval was adjusted from every three weeks to every four weeks. Subsequently, no major adverse events occurred, and follow-up CT showed shrinkage of the primary lesion, and PI did not recur.

**Fig. 3:**
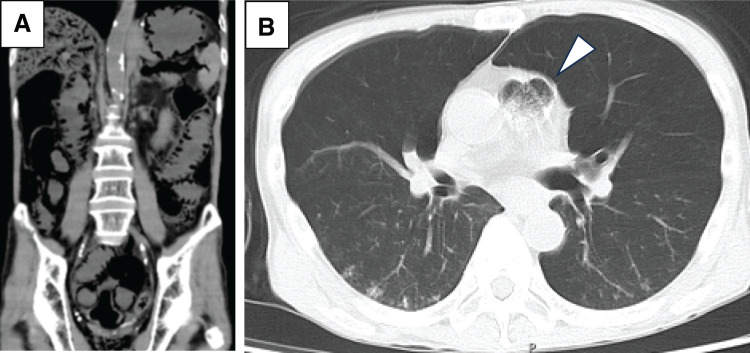
Images of CT at the second occurrence of PI and PVG. CT revealed more severe PI and PVG involving the entire length of the small intestine (**A**), with gas extending into the right atrium (arrow head) (**B**). PI, pneumatosis intestinalis; PVG, portal venous gas

Two days before initiating the fourth course (26 days after administration of the third course), blood tests showed elevated inflammation markers, with CRP level at 6.73 mg/dL and WBC at 12800/μL. As the patient did not exhibit fever, abdominal pain, or any other symptoms; therefore, a CT scan was deemed unnecessary. The following morning, the patient developed sudden-onset abdominal pain and abdominal and distension, followed by loss of consciousness and ultimately progressed to shock. Blood tests revealed further elevation of inflammatory markers (CRP 7.99 mg/dL and WBC 13200/μL). Arterial blood gas analysis revealed marked acidemia, with a pH of 7.064, base excess of −17.2, and lactate level of 18 mmol/L. CT revealed more severe PI and PVG involving the entire length of the small intestine, with gas extending into the right atrium (**Fig. 3**). Brain CT showed no visible intracranial lesions. Exploratory laparotomy was contraindicated due to the patient’s poor general condition. The patient underwent intestinal decompression and received antibiotics (meropenem) and steroids (hydrocortisone, 300 mg/day) for sepsis management. However, the patient died the following day.

**Fig. 4:**
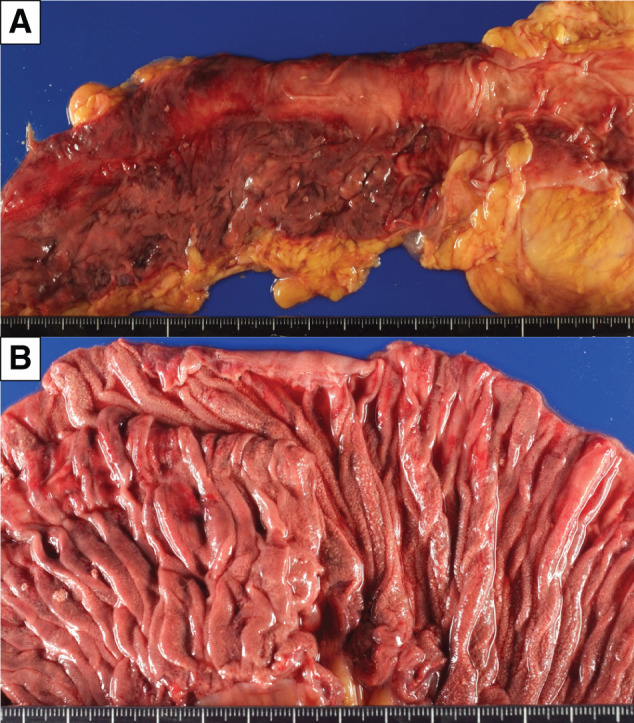
Images of macroscopic examination at autopsy. Darkly reddish features were found in cecal mucosa (**A**). Microgranular changes were present on ileal mucosa (**B**).

**Fig. 5:**
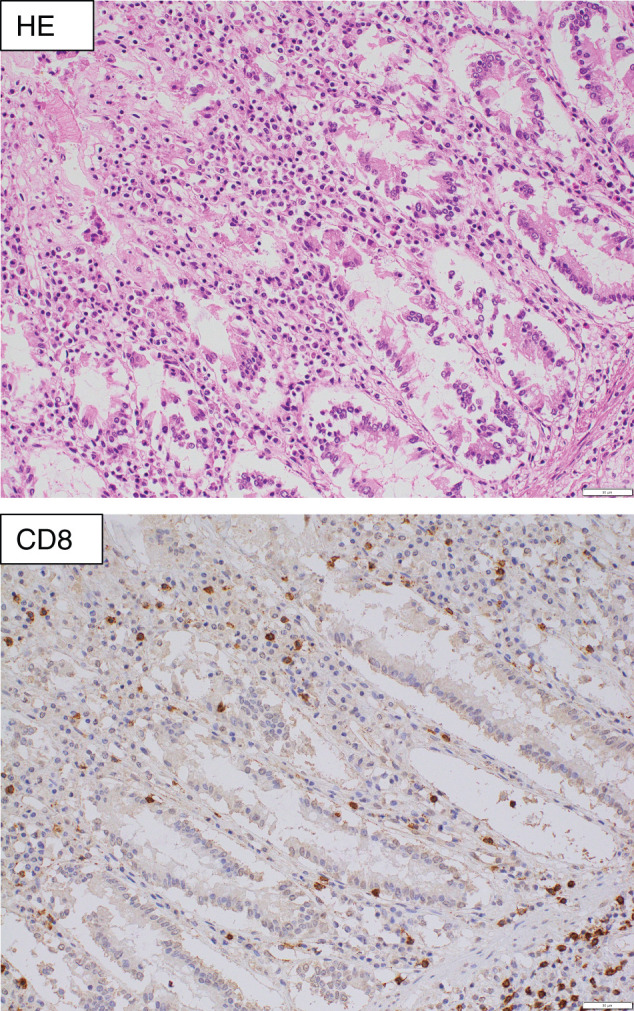
Images of microscopic examination at autopsy. Erosive cecal mucosa showed lymphatic infiltration and erosive changes. Immunohistochemically, CD8-positive lymphocytes were present in such erosive mucosa. Scales indicate 50 μm. CD8, cluster of differentiation 8; HE, hematoxylin eosin

Autopsy demonstrated segmental dark red mucosal changes of the jejunum, ileum, and colon (**Fig. 4A**), and scattered mucosal microgranular changes on the duodenum, jejunum, ileum, cecum, and ascending/transverse colon (**Fig. 4B**). Darkly reddish areas showed mucosal erosion, in which immunohistochemically CD8^+^ lymphocytes infiltrated crypts and lamina mucosae (**Fig. 5**). In mucosal microgranular areas, scattered microvacuoles with or without foreign-body reaction were present, suggesting PI. The tumor was composed mostly of viable cells, and the histological therapeutic effect was diagnosed as Grade 1a according to the Japanese Classification of Esophageal Cancer, 12th edition.^12)^

## DISCUSSION

This case report describes a patient with unresectable esophageal squamous cell carcinoma who developed two episodes of PI and PVG during pembrolizumab-based chemotherapy. The first episode occurred 3 days after the initiation of chemotherapy. It was mild and resolved within a few days after the discontinuation of enteral feeding and the use of antibiotics. By contrast, the second episode was severe and rapidly fatal. Among the various factors reported to cause PI, enteral nutrition via jejunostomy, chemotherapy with cisplatin + 5-FU, and the use of pembrolizumab were the most likely factors for recurrent PI.

Enteral tube feeding is widely used for the nutritional management of patients with gastric and esophageal cancer who have difficulty with oral intake.^13)^ However, it has been reported to cause PI.^14)^ Formula osmolarity, bacterial overgrowth, and relative intestinal ischemia are believed to contribute to PI associated with enteral nutrition.^15)^ The onset of PI associated with enteral nutrition ranges from 3 to 50 days after initiating enteral nutrition. Although most cases are mild, and improve with discontinuation of enteral nutrition, severe cases can lead to necrosis, and require surgical intervention.^14–16)^

Galm et al. reported that 5-FU induces alterations in local mucosal blood flow, and exerts both thrombogenic and vasospastic effects on the epithelium, which can lead to PI.^17)^ Cisplatin has been reported to exacerbate the gastrointestinal toxicity of irinotecan. However, no studies have suggested that cisplatin alone induces gastrointestinal toxicity.^18)^ Therefore, the potential mechanism of PI induced by cisplatin and 5-FU therapy involves a mucosal injury caused by 5-FU, which is exacerbated by cisplatin.^19)^ The onset of PI associated with chemotherapy, in the absence of molecular target drugs, ranges from six days to two months after completing a chemotherapy cycle.^19)^

In this case, moderate to severe calcification was present in the proximal superior mesenteric artery (SMA) before the onset of PI. These advanced atherosclerotic changes may have reduced mesenteric blood flow, contributing to relative intestinal ischemia. This possibility should be considered as a factor in the development of PI.^20)^

ICIs, such as pembrolizumab, have been established as promising treatment options for various malignancies. However, PIs have recently been reported as irAEs associated with ICIs.^9–11)^ According to a recent pooled analysis, irAEs generally occur between 2.2 and 14.8 weeks after initial dose.^21)^ Calenda et al. reported a case of PI occurring six months after the initiation of pembrolizumab, and two months after the most recent dose.^9)^ Similarly, Sperling et al. demonstrated that PI occurred at a median of 7 months from the first ICI dose (range: 4–17 months), or after a median of four ICI cycles.^10)^ A summary of previously reported cases of ICI-related PI is presented in **Table 1**. The table includes the timing of onset, types of ICIs, concomitant treatments, management approaches, and clinical outcomes. Unlike prior reports, our case involved two distinct episodes of PI and PVG during ICI-based chemotherapy, with the second episode resulting in death. This case underscores the importance of cautious re-challenge and the need for close monitoring. Although irAEs are characterized by their systemic onset and variability in timing, it might be unlikely that PI would develop within a few days of the first dose, given the immunological mechanism of ICIs.

**Table 1:** Summary of previously reported and present cases of ICI-related pneumatosis intestinalis

Case	ICI agent	Combined therapy	Onset	Symptoms	CT findings	Management	Outcome
1	Nivo	None	4 months	Abdominal pain	PI + PVG	Antibiotics	Recovered
2	Pembro	None	8 months	None	PI	Observation	Recovered
3	Nivo	None	5 months	Abdominal pain	PI + pneumoperitoneum	Antibiotics	Recovered
4	Pembro	Chemo	9 months	None	PI	Observation	Recovered
5	Atezo + Bev	Chemo	6 months	Abdominal pain	PI + pneumoperitoneum	Antibiotics	Recovered
6	Nivo	None	3 months	None	PI	Observation	Recovered
7	Nivo + Ipi	None	12 months	Diarrhea	PI + microperforation	Antibiotics	Recovered
8	Pembro	None	10 months	Abdominal pain	PI + pneumoperitoneum	Antibiotics	Recovered
9	Atezo	None	7 months	None	PI	Observation	Recovered
10	Nivo	Chemo	6 months	Abdominal pain	PI + pneumoperitoneum	Antibiotics	Recovered
11	Pembro	None	5 months	None	PI	Observation	Recovered
12	Durva	RT	11 months	Abdominal pain	PI + pneumoperitoneum	Antibiotics	Recovered
13	Pembro	None	6 months	Abdominal pain	PI + pneumoperitoneum	Antibiotics	Recovered
Present case							
1st	Pembro	Chemo	3 days	Abdominal pain	PI + PVG	Antibiotics	Recovered
2nd	Pembro	Chemo	2 months	Abdominal pain	PI + PVG	Antibiotics	Died

Atezo, atezolizumab; Bev, bevacizumab; Durva, durvalumab; ICI, immune checkpoint inhibitors; Ipi, ipilimumab; Nivo, nivolumab; Pembro, pembrolizumab; PI, pneumatosis intestinalis; PVG, portal venous gas; RT, radiotherapy

In this case, mucosal erosions were observed in the darkly reddish areas of the small intestine at autopsy. Immunohistochemical analysis revealed infiltration of CD8^+^ lymphocytes in the crypts and lamina mucosae. There is increasing evidence that colitis associated with irAEs is characterized by predominant accumulation of CD8^+^ lymphocytes.^22)^ By contrast, patients with inflammatory bowel disease typically exhibit higher proportions of CD4^+^ lymphocytes, regulatory T cells, and central memory T cells, along with lower proportions of CD8^+^ and CD103^+^ lymphocytes.^23)^ Takahashi et al. demonstrated that greater infiltration of CD8^+^ lymphocytes and a higher CD8^+^/CD4^+^ ratio may serve as simple and useful biomarkers for distinguishing irAE colitis from other types of colitis.^24)^ Therefore, if a biopsy had been performed via colonoscopy at the time of the first episode of PI in this case, it could have provided valuable information to guide the decision on ICI rechallenge.

At the 1st occurrence, both PI and PVG developed 2 days after initiating pembrolizumab-based chemotherapy. Considering this rapid onset, we believed that they were more likely caused by intestinal mucosal damage from enteral nutrition, or from cisplatin and 5-FU chemotherapy, rather than irAEs. However, as the number of ICI administrations increased, an increasing number of reports emerged of severe irAE-related colitis developing within just a few days.^25,26)^ Therefore, the possibility of irAE-related PI occurring within a few days after ICI administration should also have been considered. We explained the potential benefits of ICI-based chemotherapy,^2,3)^ as well as the risks associated with continuing the current chemotherapy regimen. Alternative options, including best supportive care, were also discussed. After careful consideration, the patient chose to proceed with the ICI-based chemotherapy regimen at a reduced dose. To minimize the risk of PI, preventive measures were performed, including dose reduction of cisplatin and 5-FU, the use of a fully digested formula, and the prolonged treatment interval. During the first episode, the patient showed signs of PI, but symptoms suggestive of enteritis, such as diarrhea, were mild. As a result, the chemotherapy schedule was adjusted from every three weeks to every four weeks.^27)^

The second occurrences of PI and PVG were fulminant, in contrast to initial ones. This event occurred approximately 60 days after initiating enteral feeding with a fully digested formula. No major adverse events, including PI, were observed during the second or third course of pembrolizumab-based chemotherapy. Therefore, ICI-associated irAEs were strongly suspected to be the cause of the severe second occurrence of PI and PVG, which eventually led to patient death.

This report has several limitations. First, it presents a single case, which prevents establishing a definitive cause-and-effect relationship. Second, no tests for infections such as stool culture, *Clostridioides difficile*, or cytomegalovirus were performed, as the patient underwent emergency surgery shortly after symptom onset, and no signs of infection appeared postoperatively. Third, endoscopy was not conducted during the initial episode, limiting confirmation of immune-related colitis. Fourth, the decision to resume ICI treatment was influenced by the patient’s wishes and the physician’s judgment, introducing potential bias. Finally, pathological findings were available only at autopsy, preventing evaluation of mucosal changes over the disease course.

## CONCLUSIONS

This case highlights two distinct episodes of PI during pembrolizumab-based chemotherapy for unresectable esophageal cancer. Although the exact cause of the first episode remains unclear, the fatal recurrence suggests that rechallenging with suspected causative agents poses a significant risk, even after dose reduction. While pembrolizumab-based chemotherapy was highly effective, clinicians should be aware that continuing the same treatment after an initial PI episode may be dangerous. This case underscores the importance of not only informing patients of potential risks, but also continuously reassessing the risk–benefit balance during treatment. Discontinuation of ICIs should be considered when serious irAEs are suspected. If ICI treatment is resumed after recovery from PI, close monitoring of inflammatory markers and early follow-up CT scans—even in asymptomatic patients—may facilitate earlier detection of recurrence.

## DECLARATIONS

### Availability of data and materials

Data will be made available upon reasonable request.
